# Future projections of European maize yields using AquaCrop with an adaptive growing season

**DOI:** 10.1016/j.eja.2025.127920

**Published:** 2026-02

**Authors:** Louise Busschaert, Vincent Deketelaere, Wim Thiery, Dirk Raes, Gabriëlle J.M. De Lannoy

**Affiliations:** a Department of Earth and Environmental Sciences, KU Leuven, Heverlee, Belgium; b Department of Water and Climate, Vrije Universiteit Brussel, Brussels, Belgium

**Keywords:** Yield gap, Potential yield, Water productivity, AquaCrop, Climate impact projections

## Abstract

Securing maize crop production is essential in our changing world. However, it remains unclear to what extent climate conditions and farmers’ practices, such as fertility management and irrigation, can impact future maize crop production in Europe. Here we use the AquaCrop model v7.2 in a spatially distributed setup to estimate yields, yield gaps, growing cycles, and water productivity over a 30-year baseline period (1985–2014), and a near-future period (2030–2059) under a range of climate scenarios, forced with meteorological data from the Inter-Sectoral Impact Model Intercomparison Project (simulation round 3). We define a generic maize crop with a temperature-dependent sowing date and growing stages, allowing for acclimatization of the growing cycle, in contrast to some earlier climate impact assessments. The results show that a warmer climate will lead to earlier sowing dates and shorter growing seasons, keeping future yield and yield gaps for rainfed maize relatively unchanged from the baseline. Furthermore, the area of profitable rainfed maize production may shift north and expand. In contrast to the marginal impact of climate change on near-future maize yield, removing fertility stress has the potential to increase average yields by 1.5 ton/ha (mainly in the north). An additional gain of 2 ton/ha can be obtained by optimizing irrigation in the southern regions that are not completely unsuitable for rainfed maize production. For irrigated maize in the south, the stable future yield projections are accompanied with increased water productivity, again due to an earlier and shorter growing season.

## Highlights

• Maize yield is simulated for past and future conditions using AquaCrop over Europe.
• An adaptive growing season (varying sowing date and length of growing cycle) is used.
• By midcentury, the yield (gap) will remain similar compared to that around 2000.
• Earlier sowing and harvest dates will prevent yield losses and future water stress.
• The area of biophysical profitable maize production will shift north and expand.

## Graphical abstract

**Figure:**
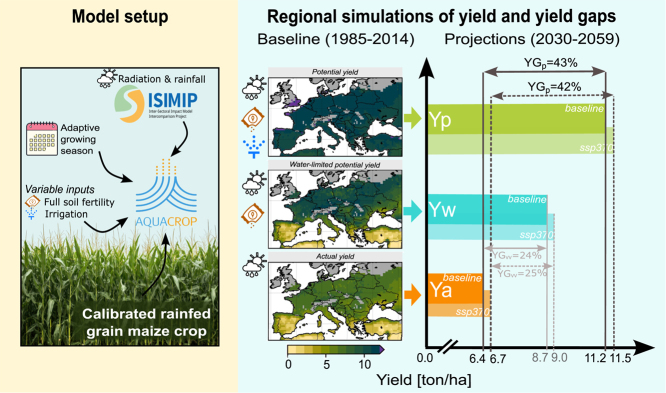

## 1 Introduction

In the coming decades, the demand for agricultural products is expected to increase due to economic and population growth (van Ittersum et al., 2013, Fukase and Martin, 2020). It is uncertain how we can meet this future demand, because climate change may shift suitable regions and periods for agriculture (Holzkämper et al., 2013). Since almost 90% (FAOSTAT, 2020) of the arable land in Europe is already being cultivated, achieving a higher yield per area is a first step toward fulfilling the future food demand. In areas with a yield gap between the actual yield and the potential yield, sustainable intensification can offer a solution (Tilman et al., 2011). This implies increasing the yields on existing croplands with proper attention to the environment and efficient use of water and nutrient resources, for instance via precision farming. Another path to sustainable intensification is to relocate the cultivation of specific crops to more suitable times and regions.

Mueller et al. (2012) presented a global assessment of yield gaps and the prospects for intensification. Their study showed that a large part of Europe currently reaches 75% of the attainable yield. Schils et al. (2018) estimated that some eastern European countries reach only 30% of their potential in cereal yield, whereas 90% is attained in western European countries. They found that further closure of the yield gap requires a large increase in crop nitrogen uptake in eastern Europe. These assessments on the continental and country scale are useful first guesses, but Europe hosts a large variety in farm structures and environmental conditions (e.g., meteorology, soils, fertilizers, pesticides) and thus a spatial range of possible output (crop yield). Furthermore, the yield potential is likely to change in time with climate change, agricultural innovation and adaptive farming practices. It is therefore crucial to develop realistically parameterized spatial crop modeling systems to produce spatiotemporally varying yield estimates, and to design future climate impact simulations that are valuable for decision making purposes.

Maize (*Zea mays L.*) is an important summer crop used for applications from human food and fiber needs to animal feed and energy production. In Europe, the average area used for maize production in the last 5 years (2019/20 - 2023/24) is 8908 ⋅10${}^{3}$ ha with a production of about 64 ⋅10${}^{6}$ ton, or 7.2 ton/ha (USDA, 2025b.). The question is where and by how much Europe could possibly increase its production to meet future demands for grain maize, and how this is affected by climate change. A related question is where in Europe a cost-effective production of maize could or could not be supported in the future, given the projected supplies of water, CO${}_{2}$ and heat, or investments in irrigation and fertilization.

**Fig. 1:**
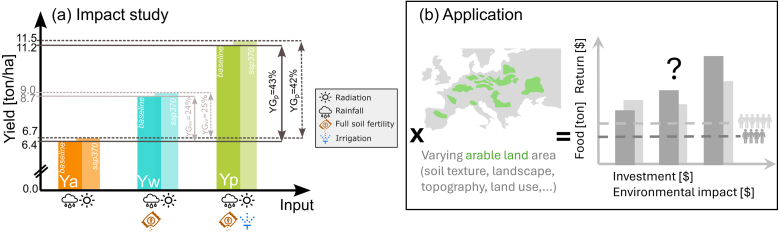
(a) Concept of actual, water-limited and potential yield ($Y_{a}$, $Y_{w}$, $Y_{p}$), and yield gaps ($YG_{w}$, $YG_{p}$), for the (dark) baseline and a (light) future climate scenario (SSP3-7.0). The values are spatial means of 30-year averages over the crossmasked area where rainfed $Y_{a}$ could hypothetically be above 4 ton/ha on average in Europe, without accounting for the current or future arable land area. Details are explained in Section 3.3. (b) Example of socio-economic application: by limiting the theoretical yield estimates to the arable area in Europe, estimates of total food supply and financial return could be compared to the population-driven demand (not in this paper).

In this paper, we address these questions by performing and analyzing historical and future simulations of spatially distributed actual farmer’s yield ($Y_{a}$) and potential yield of maize, either without or with water limitations. The concept is illustrated in Fig. 1a. Potential yield ($Y_{p}$) is the maximum attainable yield in the absence of water or nutrient limitations, or other biotic stresses. Only energy limitations constrain the $Y_{p}$, and the latter is therefore location specific (solar radiation, temperature). $Y_{p}$ serves as a benchmark in areas where water availability is no limitation, e.g. in humid or irrigated areas. By contrast, water-limited yield ($Y_{w}$), or water-limited potential yield, accounts for limited water availability to the plants, and is the most relevant benchmark for rainfed crops. The gap between the potential yield ($Y_{p}$ or $Y_{w}$) and the actual or farmer’s yield ($Y_{a}$) can be reduced by agricultural management. To quantify crop production in relation to water supply, availability or use, various concepts have been defined (Kumar, 2021, Bacon, 2004). We define the water productivity WP (also sometimes referred to as water use efficiency) as the agricultural yield (output) per unit of water lost by evapotranspiration on the field (input). The actual and potential water productivity are considered.

Yield gaps and (potential) water productivity can be determined through field experiments, but even in the most controlled environment, it remains difficult to ensure that all stresses are avoided for the estimation of $Y_{p}$. Therefore, crop models are often used to simulate ideal conditions and then derive yield gaps at the field scale or regionally around reference weather stations (Grassini et al., 2015, Aramburu Merlos et al., 2015, Farmaha et al., 2016, Silva et al., 2023). However, in a global economy, a geospatially explicit quantification of the exploitable yield gaps under present and future conditions is needed (van Ittersum et al., 2013). This is complex, because large-scale long-term consistent databases of spatially distributed $Y_{a}$ are hardly available as a reference and the large-scale spatially distributed application of process-based crop models is still in its infancy (Pasquel et al., 2022). Large-scale crop simulation efforts, such as the Agricultural Model Intercomparison and Improvement Project (AgMIP) Global Gridded Crop Model Intercomparison (GGCMI; Jaegermeyr et al., 2021), are increasingly supported by digital soil maps, various sources of weather data and climate projections, satellite-based irrigation maps, crop maps and crop calendars, even if the latter are often static in time. However, poor assumptions are often made about, for instance, crop parameters or start and end of the growing season, leading to different model sensitivities to climate drivers (Müller et al., 2024) and a risk of biasing the crop response in climate studies. For example, Jägermeyr and Frieler (2018) stated that better information about the growing season is needed in global models to reproduce the impacts of extreme events, and Minoli et al. (2022) highlighted that adaptive management of crop growth periods can reduce the negative impacts of climate change and enhance the positive CO${}_{2}$ fertilization effect.

This study will address the above concerns by employing spatio-temporally variable sowing dates and growing cycles for maize. Given that sowing dates and growing cycles vary both with latitude and across the years, we assume that this variability will respond to climate, and is thus crucial for climate studies. The approach is based on the experience of agronomists and differs from studies that used a fixed crop cycle length and calendar for Europe in large-scale crop model experiments (Elliott et al., 2015, Müller et al., 2019, de Roos et al., 2021, Busschaert et al., 2022, Jaegermeyr et al., 2021). Although we assume an earlier start of the growing season and a shorter season in warmer climates, literature on the start and end of the maize growing season within Europe is diverging: Sacks et al. (2010) and Jaegermeyr et al. (2021) show a latitudinal pattern in the sowing date, but there is no such pattern in the length of the growing season in the crop calendar information used for GGCMI (Jaegermeyr et al., 2021), and some studies report longer stage lengths for fields in southern European countries than in northern countries (Allen et al., 1998, Katerji et al., 2013, Yang et al., 2017, Schils et al., 2018). One reason for this discrepancy may be that the above studies map various maize variants across Europe (also irrigated), or consider maize as e.g. a second crop in a larger cropping system, whereas we only consider one cycle of maize production. For climate studies that focus on relative changes, we believe that it is essential to (i) simulate a flexible crop growth to capture the impact of temperature (adaptive growing season), and (ii) assume a fixed crop variant and hinge the conclusions on it, or else specify how the variant changes in time, but the latter is unknown.

In addition to the information on growing cycle and planting dates, many crop models require detailed information which is simply not available at the large scale. The crop-water model AquaCrop (Steduto et al., 2009, Raes et al., 2023) offers a solution, because it asks for relatively few inputs but remains complex enough to accurately simulate crop growth. AquaCrop is increasingly used for regional applications (Lorite et al., 2013, Han et al., 2020, de Roos et al., 2021, Busschaert et al., 2022, Mialyk et al., 2022, de Roos et al., 2024, Busschaert et al., in review), and to assess climate scenarios for crop production and irrigation needs (Busschaert et al., 2022). The latter typically use generic crops with phenological stages defined on fixed calendar days. Climate scenarios specifically for maize yield with AquaCrop have so far been limited to particular field sites or small regions (Yang et al., 2017, Durodola and Mourad, 2020, Raes et al., 2021).

This study uses the AquaCrop model v7.2 in a spatially distributed setup (de Roos et al., 2021, Busschaert et al., 2022) over Europe, with a temperature-dependent definition of grain maize crop parameters, and forced with climate data from the Inter-Sectoral Impact Model Intercomparison Project simulation round 3 (ISIMIP3; Frieler et al., 2024). The objectives are to (i) quantify the yield gap and water productivity of a generic European grain maize cultivar for the current and future climate, (ii) identify which regions in Europe will become more or less suitable for grain maize production under a future climate, and (iii) demonstrate to which extent investments in irrigation and field management (e.g., fertility) can help crop production, while assuming an unchanged maize cultivar. An important difference between this and other climate impact studies is that all simulations include a dynamic computation of the sowing dates and growing seasons, implying a form of climate adaptation.

## 2 Material and methods

### 2.1 Study area

With 5% of the global grain maize production, the European Union is the fourth largest producer, after the US, China and Brazil (USDA, 2025a). Therefore, our study area consists of a large part of the European continent, including the current main producing areas for rainfed grain maize and surrounding areas. The model simulations are performed over a grid from 34.75°N to 59.75°N latitude and -10.75°E to 41.25°E longitude at 0.5°spatial resolution, aligned with the grid of the meteorological input (Frieler et al., 2024). Note that the simulations are hypothetical and that all output is expressed by unit area (ton/ha). We do not account for the current or future available arable land area, which would be needed to derive total amounts (ton) of production over Europe for socio-economic applications (Fig. 1b).

### 2.2 AquaCrop model setup

AquaCrop is a state-of-the-art crop model that offers a good balance between simplicity, accuracy, and robustness (Steduto et al., 2009, Raes et al., 2009). AquaCrop focuses on a water-driven simulation of biomass and yield, whereas most other crop models are rather carbon- or solar-driven (Bouman et al., 1996, Brisson et al., 2003, Jones and Kiniry, 1986, Jones et al., 2003, Ritchie et al., 1985, van Ittersum et al., 2003). In many agricultural areas in our study domain, water availability is indeed a dominant limiting factor during the growing season. Other limiting factors are pest control or nutrients (e.g., nitrogen, phosphorus), or even post-harvest loss, which are hard to quantify. For the latter, AquaCrop defines a general bulk estimate of the effect of field management on crop production through a corresponding ‘soil fertility stress’ (Van Gaelen et al., 2015). Note that unlike unofficial AquaCrop versions, the official Fortran-based AquaCrop v7.0 and higher versions include the simulation of fertility stress. The model simulations are performed using a Python wrapper (de Roos et al., 2021, Busschaert et al., 2022) to call AquaCrop v7.2 in a spatially distributed way, for each 0.5°grid cell.

Following the setup of Busschaert et al. (2022), the simulations are forced with ISIMIP3 climate input, which consists of near-surface temperature, precipitation, near-surface relative humidity, 10-m wind speed, shortwave downwelling radiation, and atmospheric CO_2_ concentration. The reference evapotranspiration ET${}_{o}$ is calculated using the FAO Penman-Monteith guidelines described in Allen et al. (1998), based on the daily variables available in the ISIMIP3 dataset. AquaCrop is using the FAO-56 dual crop coefficient approach (Allen et al., 1998) and its particularity is that the dual crop coefficient ($K_{cb}$) is not prescribed as fixed values through the initial, mid-season, and end $K_{cb}$ values, but it is dynamically determined based on the simulated canopy cover and a crop coefficient parameter for when the canopy is fully developed (canopy cover is 1), $K_{c,Tr,x}$. The soil evaporation is also dynamically adjusted with the canopy cover and depends on the maximum soil evaporation coefficient which is a program parameter. More information on AquaCrop calculation procedures can be found in Raes et al. (2023).

Soil input data is extracted from the latest version ISIMIP3 soil dataset, which is based on the Harmonized World Soil Database version 1.2 (HWSD1.2) and aggregated to 0.5°, also used for the GGCMI (Rosenzweig et al., 2014). Only the dominant soil types over croplands are represented. The hydraulic parameters are derived using pedotransfer functions as in De Lannoy et al. (2014). More details can be found in Busschaert et al. (2022).

A generic European grain maize cultivar is parameterized in a ‘crop file’ that defines crop characteristics related to growth, phenology, and stress (see Appendix A and Table A.1 for a full explanation). One crop file with stage parameters in growing degree days (GDDs [°C], see Section 2.7) is used for the entire study domain, assuming that farmers keep maize on the fields for a similar number of GDDs. Furthermore, a temperature-dependent sowing criterion is used. These aspects are further elaborated in Section 2.7. The crop features are assumed to remain the same in the future because the technological advances remain uncertain (as discussed in Section 4.3.4).

The ‘soil fertility stress’ on crop production is set in the ‘management file’ of AquaCrop, and parameterized based on reference data discussed in Section 2.3. Specifically, a uniform soil fertility stress of 30% is assumed over the entire study domain. Furthermore, it is assumed that farmers will want to maintain this relative high field management (sound soil fertility management and control of pests and diseases) also in the future.

### 2.3 Simulations for model calibration

To tune the maize crop and fertility parameters, AquaCrop simulations of actual and water-limited potential yield ($Y_{a}$, $Y_{w}$) are forced with reanalysis-derived meteorological input and compared with reference yield data for rainfed maize from the Global Yield Gap Atlas (GYGA; GYGA, 2021) over the current maize production area, for the period 2011 through 2016. The meteorological data are based on bias-adjusted ERA5 reanalysis data for the period 1979–2019 (GSWP3-W5E5). The GSWP3-W5E5 product is the main meteorological forcing dataset within ISIMIP3a and provides daily data at 0.5°spatial resolution.

The GYGA $Y_{a}$ observations are largely based on statistical surveys, are not available for all years, and do not account for the spatio-temporal variability of the reporting fields within a country. The corresponding GYGA $Y_{w}$ estimates are obtained from crop model simulations at sites with weather data (Grassini et al., 2015), but these sites are not necessarily representative of the same times and locations as those used for GYGA $Y_{a}$. Therefore, the AquaCrop simulations are also not extracted over any specific maize fields, but instead extracted as the average of the cells in each country, and averaged over 6 simulation years (when enough GDDs were available to complete the growing cycle). The performance of the yield simulations is summarized through three skill metrics computed spatially (over the different countries): the Pearson R [-], the bias (GYGA Y - AquaCrop Y) [ton/ha], and the root mean square difference (RMSD) [ton/ha].

The soil fertility stress is determined based on the GYGA $Y_{a}$ and $Y_{w}$ yield statistics. The ratio of $Y_{a}$ over $Y_{w}$ corresponds to the relative yield (or biomass) that can be obtained compared to the potential. Over the study area, this ratio is 70% on average (relative biomass production $B_{rel}$ of 70%, corresponding to 30% fertility stress), corresponding to a moderate to near optimal biomass production.

AquaCrop is thus calibrated for rainfed maize at mid-latitude regions only using $Y_{w}$ (and $Y_{a}$ to derive the degree of soil fertility stress), whereas the $Y_{p}$ simulations are not calibrated. Therefore, the results will focus on rainfed maize. However, relative changes in AquaCrop $Y_{p}$ will be considered in the southern countries as an indication of how climate change might impact the irrigated yield and associated variables. The choice was made to mainly focus on rainfed maize because the expansion of irrigated areas remains uncertain in the future (Hurtt et al., 2020, Schaldach et al., 2012) and considering this change would add another layer of uncertainty.

### 2.4 Simulations for climate impact assessment

The calibrated AquaCrop model is used to estimate yields, yield gaps, growing cycles and water productivity averaged over a 30-year baseline period of 1985–2014, and a near-future period of 2030–2059, both forced with climate model output available through ISIMIP3b. The goal is to quantify the change in the 30-year averaged yield (and other variables) for the future scenarios relative to that of the baseline for the entire study domain. Whereas ISIMIP3a provides historical re-analysis climate data, ISIMIP3b provides estimates of both historical and future climate (ISIMIP3b), following different emission scenarios and using various bias-adjusted global climate models (GCMs). All (baseline and future) AquaCrop simulations are performed with climate input from 5 GCMs (GFDL-ESM4, IPSL-CM6A-LR, MPI-ESM1-2-HR, MRI-ESM2-0, and UKESM1-0-LL; Lange, 2019, Lange et al., 2020) to account for some of the model uncertainty, and the ensemble median values are used to produce the results, except in Section 4 where the difference between the GCMs is analyzed.

For the baseline CO_2_ concentration, AquaCrop uses a historical dataset of yearly observations at the Mauna Loa Observatory (Hawaii, USA). For the future climate scenarios, we consider ISIMIP3b projections (Frieler et al.) associated with Shared Socioeconomic Pathways (SSPs) SSP1-2.6, SSP3–7.0, and SSP5–8.5, where the digits after the dash indicate the representative concentration pathway (RCP). Because the warmest available climate scenario (SSP5-8.5) is unlikely to happen, the focus is on SSP3-7.0, even if this is a distinct scenario (Shiogama et al., 2023). The selected scenario has shown value in providing a reference for a world that fails to cooperate on climate action and experiences high emissions (Fujimori et al., 2017). The results for other pathways are included in Section 4.

A strong assumption in our simulation set-up is that field management (fertility) and crop characteristics of maize will not change in the future. However, our parameterization of crop growth stages in terms of GDDs renders the growing cycles responsive to future temperature regimes. For example, a warmer climate will lead to earlier sowing dates and shorter growing seasons. Furthermore, future change in water availability will affect growth stresses. Also, increased CO${}_{2}$ concentrations will increase biomass production in the absence of soil water and fertility stress (Vanuytrecht et al., 2012). However, the photosynthetic pathway (C4) of maize makes it theoretically (and through its implementation in AquaCrop) less responsive to elevated CO${}_{2}$ concentrations than C3 crops (e.g. wheat, rice), although not all studies support this theory (Wand et al., 1999). Other climatic effects such as losses from heat stress, wind throw, wildfires, pest outbreaks, false spring, or flooding, are not included in this study but their effects are discussed in Section 4.3.4.

### 2.5 Yield and yield gap

The various types of yield and yield gaps are defined in Fig. 1a. The actual farmers’ yield (or simply actual yield), $Y_{a}$ [ton/ha], for a specific cultivar is the yield that is limited by water availability (weather, soil), and additionally limited by factors such as soil fertility, weed management and crop diseases (van Ittersum and Rabbinge, 1997). The AquaCrop $Y_{a}$ simulations are obtained by setting the fertility stress at 30% ($B_{rel}$ of 70%). The rainfed yield potential $Y_{w}$ [ton/ha], is a yield that is also limited by water supply, but there are no other limitations. The AquaCrop $Y_{w}$ simulations are obtained by setting the fertility stress to 0% ($B_{rel}$ of 100%). The yield potential, $Y_{p}$ [ton/ha] is the yield under non-limiting conditions: there is enough water and nutrients, and biotic stresses are absent. It is the highest possible attainable yield, solely controlled by cultivar, radiation, temperature and CO${}_{2}$ concentration. The AquaCrop $Y_{p}$ simulations are obtained by setting the fertility stress to 0% and activating irrigation in such a way that the root-zone soil moisture is kept above 50% of the readily available water (field capacity - moisture content when stomatal closure starts). In all cases, the simulated yield refers to the dry grain yield.

The yield gap can be used to identify regions where the actual yield can be significantly increased by overcoming some or all limitations. The water-limited yield gap ($YG_{w}$) [%] and the potential yield gap ($YG_{p}$) [%] are defined as follows:

(1) $$YG_{w}=\left(1-Y_{a}/Y_{w}\right).100$$

(2) $$YG_{p}=\left(1-Y_{a}/Y_{p}\right).100$$

### 2.6 Water productivity

The water productivity ($WP_{ET}$) is defined as the ratio of economic yield of a crop per unit of crop evapotranspiration (ET${}_{c}$), defined in kg yield per m${}^{3}$ water evapotranspired. It is an indicator of how efficiently water is used, also referred to as crop water use efficiency. $WP_{ET}$ is computed by dividing the end-of-season yield by the sum of the daily ET${}_{c}$ during the growing cycle (i.e. varying length from germination to crop maturity). For each definition of yield, there is a corresponding $WP_{ET}$, i.e. potential ($WP_{p,ET}$), water-limited ($WP_{w,ET}$), and actual ($WP_{a,ET}$), and an associated $WP_{ET}$ gap. The $WP_{ET}$ in AquaCrop responds non-linearly to the water supply and atmospheric CO${}_{2}$ concentrations.

### 2.7 Temperature-dependent sowing date and growing cycle

The crop stages are defined in GDD and the sowing date is temperature-dependent for all simulations. The temperature-dependency makes the sowing date and the growing cycle sensitive to a change in climate conditions, unlike many other climate sensitivity studies (see below). The growing cycle is defined from germination to crop maturity.

GDDs measure heat accumulation over time, which directly influences crop development. The GDD is calculated as GDD = T${}_{av}$ - T${}_{base}$, with T${}_{av}$ the average day temperature and T${}_{base}$ a crop-specific base temperature, below which no crop development occurs. The latter a conservative parameter in AquaCrop and is set at 8 °C (Hsiao et al., 2009). Temperatures above a crop-specific upper value T${}_{upper}$ of 30 °C (Hsiao et al., 2009) are not included in the GDDs, because excessive temperatures do not contribute to crop development. Simulations in GDD mode can better account for temperature effects on crop growth and development than simulations in calendar days. The GDDs were manually calibrated and constrained by agronomist expertise, with the objective of providing reasonable yield estimates in accordance with the GYGA data (Section 2.3). In some regions (northern or high altitude) and for some years, the temperature values might not be high enough to accumulate enough GDDs to complete the maize growth cycle as defined by the crop parameters. The grid cells with less than 5 years of complete growth cycle (i.e., enough GDDs) are excluded from the analysis for the future yield simulations (Section 2.4), but all years are considered for the model calibration (Section 2.3).

The temperature regime not only defines the length of the crop development stages, but also defines the start of the growing season through the sowing date. Specifically, the sowing date is the first day between 1 March and 29 April (60 days range) where the minimum temperature is above 8 °C for the last 4 days. If this criterion is not met, the sowing date is set to 29 April (last day of the window), based on reported dates of maize emergence and sowing (Schils et al., 2018, Franch et al., 2022) and agronomist expertise. This temperature criterion is used to ensure that the soil temperature is warm enough for seed germination, which is around 8 - 10 °C (Sacks et al., 2010). If field conditions allow an early sowing, a simulation in GDD can lead to a longer growing cycle, if the spring temperatures are low and the canopy growth is slow, e.g. in northern countries. For a generally warmer climate, the sowing date may be earlier and the growing season shorter (i.e., more GDD).

## 3 Results

### 3.1 Model calibration

**Fig. 2:**
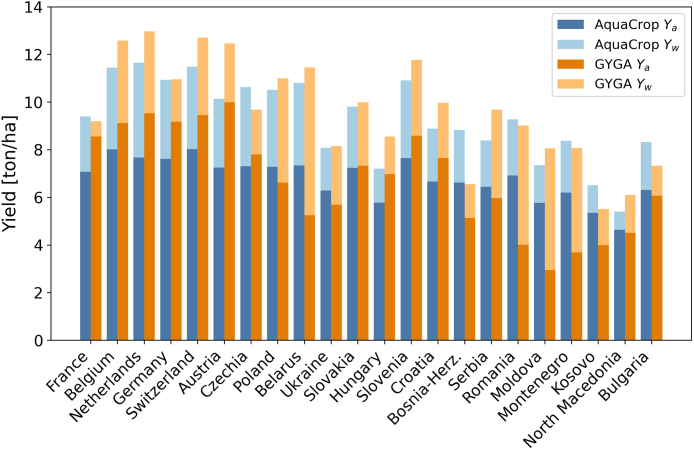
Comparison of AquaCrop simulations against GYGA estimates of 6-year averaged $Y_{a}$ and $Y_{w}$ for 2011–2016. $Y_{w}$ yield values are stacked on top of $Y_{a}$ values for countries with GYGA data ordered from west to east.

AquaCrop simulations for rainfed maize are tuned toward GYGA $Y_{a}$ observations and GYGA $Y_{w}$ simulations for the years 2011–2016. Fig. 2 shows a gradient in the GYGA $Y_{a}$, with values between 3 ton/ha in the east and up to 9 ton/ha in the west of Europe. AquaCrop roughly follows the pattern but with slightly underestimated yield in the west, and overestimated yield in the east, likely due to an assumed constant fertility stress. In general, AquaCrop $Y_{a}$ in Europe is within the observed range of GYGA values. By design, the simulated $Y_{w}$ is always higher than $Y_{a}$, because fertility stress is eliminated. However, water stress is still included and drives similar spatial patterns for AquaCrop and GYGA $Y_{w}$.

Overall, the AquaCrop yield simulations show a reasonable performance with a Pearson R of 0.74, a bias of −0.06 ton/ha, and an RMSD of 1.58 ton/ha for $Y_{a}$. For $Y_{w}$, the Pearson R (0.89) and RMSD (1.07 ton/ha) show a slightly better performance in comparison to $Y_{a}$, but with a mean underestimation of 0.34 ton/ha.

Note that the $Y_{a}$ estimates are only for rainfed maize, and we will primarily discuss the impact of fertility, irrigation and climate change on rainfed maize. For irrigated maize, the climate impact on $Y_{p}$ will be considered but should be taken only as a relative change, since the crop was not properly calibrated for irrigated conditions.

### 3.2 Climate projections

**Fig. 3:**
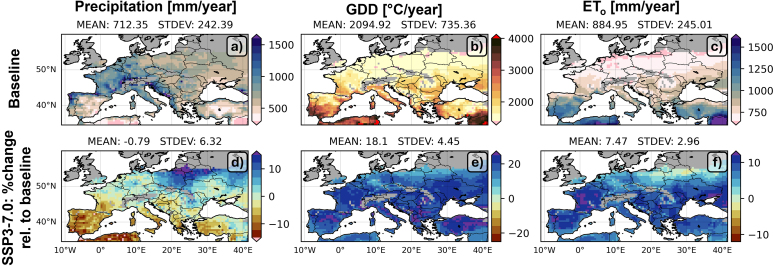
30-year averaged (top row) annual baseline climate variables and (bottom row) difference between SSP3-7.0 (2030–2059) and the baseline (1985–2014), relative to the baseline [relative change, %]: (a,d) annual precipitation, (b,e) annual GDD, (c,f) annual ET${}_{o}$. Gray cells mark locations where not enough GDDs could be accumulated to fulfill the baseline maize crop growth cycle.

The SSP3-7.0 climate projections are associated with an elevated CO${}_{2}$ concentration, a change in precipitation patterns, and higher temperatures. The 30-year average (1985–2014) annual baseline climate and the relative change for the near-future period (2030–2059) under the SSP3-7.0 scenario, are shown in Fig. 3. For each grid cell, the median value across the 5 GCMs is shown. The median is chosen over the mean because it offers more robustness for a small ensemble. For a grid cell to be included, there should be at least 5 years of completed crop cycle in all GCMs, that is, with enough GDDs (1122 °C, see Appendix A) from sowing to the end of the calendar year. The baseline patterns show slightly more precipitation in the northwest than the south and east. Note that the available precipitation during the growing season is less than the annual values plotted here. The average annual number of GDD is about 2095 °C/year. The ET${}_{o}$ shows an inverted pattern of precipitation, with a high atmospheric demand for water in the south and lower values in the north. For SSP3-7.0, slightly more precipitation is expected in the northeast and less in the southern part of Europe. The temperature increases throughout Europe, resulting in a 18% increase in GDDs in Europe relative to the baseline. SSP3-7.0 also leads to a 7% increase in ET${}_{o}$, due to an increase in radiation and vapor pressure deficit. This will drive an increase in biomass productivity. The baseline and future patterns of the climate variables corroborate with earlier studies (e.g., IPCC report; Bednar-Friedl et al., 2022).

Part of the British Isles, Scandinavia and the Alps cannot accumulate enough GDDs in the baseline simulation to fulfill the maize crop growth cycle (Fig. 3b). Not shown (because values are expressed relative to the baseline) is that less area suffers from this shortcoming in future (warmer) climate scenarios, as will be discussed in Section 4.1. The combination of changes in average climate and in the temporal variability of climate variables in SSP3-7.0 determine the crop simulations described in the next section.

### 3.3 Yield and yield gap under historical and future climate conditions

**Fig. 4:**
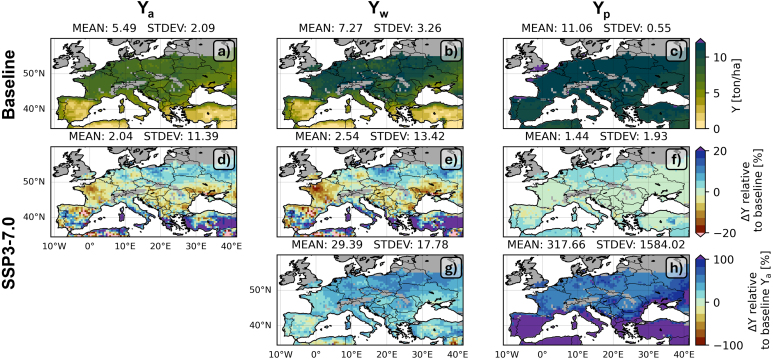
30-year averaged (top row) baseline $Y_{a}$, $Y_{w}$ and $Y_{p}$, (second row) difference between SSP3-7.0 (2030–2059) and baseline (1985–2014) $Y_{a}$, $Y_{w}$ and $Y_{p}$ relative to the respective baseline estimates [relative change, %], (third row) same as second row, but now relative to baseline $Y_{a}$ [relative change, %].

Fig. 4a shows the 30-year average (1985–2014) baseline $Y_{a}$ for the median across the GCMs at each pixel in the study domain. These simulations are forced with the ISIMIP3b GCMs and in line with those in Fig. 2, where AquaCrop is forced with reanalysis data from ISIMIP3a and evaluated for selected countries during the period 2011–2016. $Y_{a}$ is limited by fertility and water availability, and amounts to about 5.5 ton/ha across the entire study area, including the southern regions where no rainfed maize is cultivated, and excluding the high latitude and altitude areas where not enough GDDs are accumulated. When excluding the southern regions with average yields below 4 ton/ha to focus on the rainfed maize production area, the spatial mean is 6.4 ton/ha (Fig. 1a).

**Fig. B.1:**
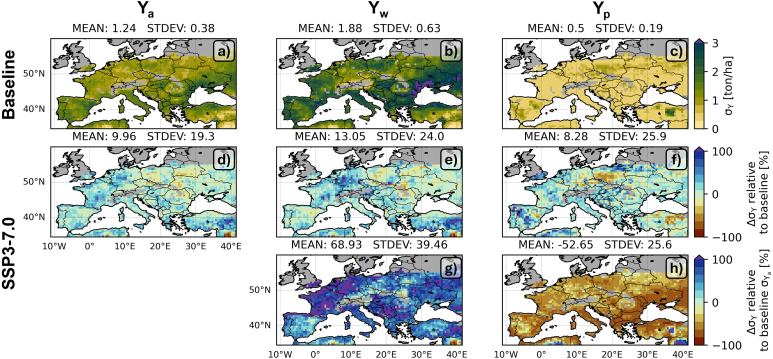
Same as Fig. 4 but for 30-year standard deviation $Y_{a}$, $Y_{w}$ and $Y_{p}$.

By removing fertility limitations, the water-limited dry yield $Y_{w}$ increases to about 7.3 ton/ha (8.7 ton/ha, excluding the southern regions), with primarily increased $Y_{w}$ for the northern latitudes (Fig. 4b). Since $Y_{w}$ remains close to $Y_{a}$ for the drier regions (south), the spatial variability is higher for $Y_{w}$ compared to $Y_{a}$. When irrigation is introduced and, thus, both the water and fertility limitations are removed, the potential dry yield $Y_{p}$ is 11 ton/ha with little spatial variability (Fig. 4c). Irrigation mainly contributes to high $Y_{p}$ in the southern regions which are known to be water-limited (e.g., Teuling et al., 2009, Thiery et al., 2017). The $Y_{p}$ values in the Mediterranean countries agree well with the GYGA $Y_{a}$ of irrigated maize, and are slightly less than the simulated GYGA $Y_{p}$ (not shown). The temporal standard deviation across the 30 years is about 1 ton/ha for $Y_{a}$, 2 ton/ha for $Y_{w}$, and only 0.5 ton/ha for $Y_{p}$ (Fig. B.1a, b, c).

Under SSP3-7.0 and considering the same area as the baseline, the $Y_{a}$, $Y_{w}$ and $Y_{p}$ remain similar to the baseline, but $Y_{a}$ and $Y_{w}$ slightly increase for the northern latitudes and Mediterranean areas where the baseline values are low (Fig. 4d,e,f). The northern latitudes benefit from a combined increase in temperature and precipitation. The southern latitudes gain from an earlier sowing date and shorter growing season which avoids severe water stress later in the summer, and an earlier harvest which allows to replenish the soil water. Furthermore, the elevated CO${}_{2}$ concentrations will also partially limit yield losses that could be expected from increased water stress in southern regions (Fig. 3d). For SSP3-7.0, some local $Y_{a}$ loss is found in e.g. northwest France (Fig. 4d), but removing the fertility stress alone can bring the future yield back to the baseline $Y_{a}$ and even to on average 30% higher values (Fig. 4g), although with a large relative increase in temporal variation within the 30 years (Fig. B.1g). Overall, SSP3-7.0 increases the interannual variation in yields by about 10 % (slightly more for $Y_{w}$, less for $Y_{p}$, Fig. B.1d, e, f) relative to the baseline.

**Fig. 5:**
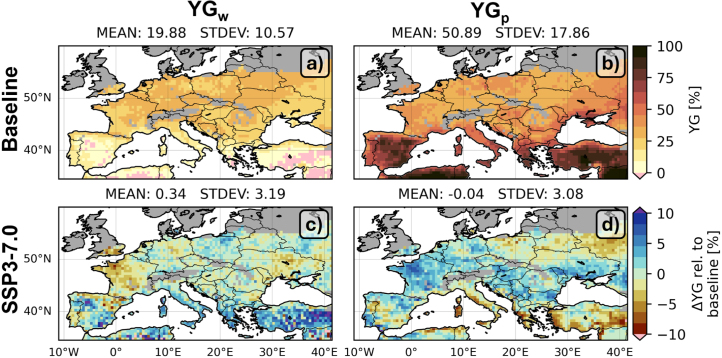
30-year average (top row) baseline $YG_{w}$ and $YG_{p}$, (second row) difference between SSP3-7.0 and baseline (a, (c) $YG_{w}$ and (b, (d) $YG_{p}$, relative to the respective values for the baseline [relative change, %].

Fig. 5 shows that the baseline $YG_{w}$ and $YG_{p}$ have a latitudinal variation. The baseline $YG_{w}$ is very low in the southern countries (Fig. 5a), where water - and not fertility - is the main limitation, and it is about 30%–40% in the central to northern regions. This is a design feature, i.e. 30% of fertility stress was assumed in $Y_{a}$ for rainfed maize. In some areas where rainfed maize production is impossible, $YG_{w}$ is even negative, because the $Y_{w}$ is lower than $Y_{a}$: abundance of fertility allows the crop to develop and increase its transpiration demand, which may not be met, leading to a lower $Y_{w}$ (Raes et al., 2021). The $YG_{p}$ ranges between 30 and 50% in mid-latitude Europe, and goes up to 100% in southern Europe, where water is indeed the main limitation (Fig. 5b). For SSP3-7.0, $YG_{w}$ remains similar to the baseline, but the negative $YG_{w}$ is overcome (Fig. 5c). $YG_{p}$ also remains similar to the baseline (Fig. 5d), and areas with an increase or decrease in $YG_{w}$ for SSP3-7.0 typically feature the opposite change in $YG_{p}$. For example, in northwest France, both $Y_{a}$ and $Y_{w}$ decrease, but $Y_{w}$ decreases relatively more than $Y_{a}$ leading to a reduced $YG_{w}$ for SSP3-7.0. By contrast, $Y_{p}$ remains stable, leading to an increase in $YG_{p}$ in this area.

### 3.4 Growing cycles and water productivity under historical and future climate conditions

**Fig. 6:**
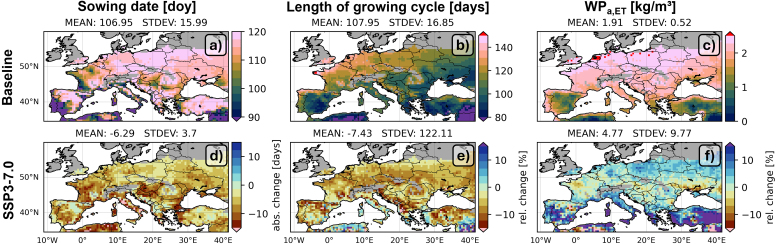
30-year averaged estimates of the sowing date, length of the growing cycle (germination to maturity) and water productivity ($WP_{a,ET}$) under actual farmer conditions, and without future adaptations, for (top) the baseline, and (bottom) difference between SSP3-7.0 and the baseline, relative to the respective values for the baseline period [relative change, %].

The differences between the baseline and SSP3-7.0 simulations are associated with differences in the growing cycle. Fig. 6a shows that the sowing date for the baseline varies from the first week of March to the end of April for rainfed maize: the model is parameterized to start no later than 29 April. The southern countries have an earlier sowing date than the northern countries, in line with some static crop calendars (Sacks et al., 2010, Jaegermeyr et al., 2021). Fig. 6d illustrates that the warmer temperatures for SSP3-7.0 allow to start the growing season on average 6 days earlier than in the baseline simulation, with a shift of more than 2 weeks for the southern regions. At the same time, the growing cycle is generally shortened under SSP3-7.0, by a week on average. Note that the growing cycle is particularly shortened in areas where the sowing date is not pushed to an earlier date (start later in the season, warmer temperatures, more GDD), and it is not much shorter when the sowing date is earlier (lower temperatures). The earlier sowing dates and shorter growing cycles contribute to avoiding crop yield losses that would be expected from water stress in SSP3-7.0.

Finally, the baseline water use efficiency or water productivity $WP_{a,ET}$ is about 2 kg/m${}^{3}$ across the study domain, but with values below 1 kg/m${}^{3}$ in Mediterranean countries (assuming no irrigation) (Fig. 6c). Not shown is that the average $WP_{w,ET}$ and $WP_{p,ET}$ reach up to 2.5 and 3 kg/m${}^{3}$ across the study domain, where fertility leads to high $WP_{w,ET}$ for northern latitudes, and the additional hypothetical unlimited water supply through irrigation results in high $WP_{p,ET}$ in southern regions. These values are in line with field measurements (Zwart and Bastiaanssen, 2004). For SSP3-7.0, $WP_{a,ET}$ slightly increases on average: there are small decreases in the central latitudes due to water stress, and increases in $WP_{a,ET}$ for the northern and southern latitudes (Fig. 6f) because of more precipitation and GDD, and earlier planting, respectively. Furthermore, reductions in $WP_{a,ET}$ are prevented by CO${}_{2}$ fertilization. The patterns of relative change are similar for $WP_{w,ET}$ and $WP_{p,ET}$ (not shown).

## 4 Discussion

### 4.1 Shift in dominant maize production area and period

**Fig. 7:**
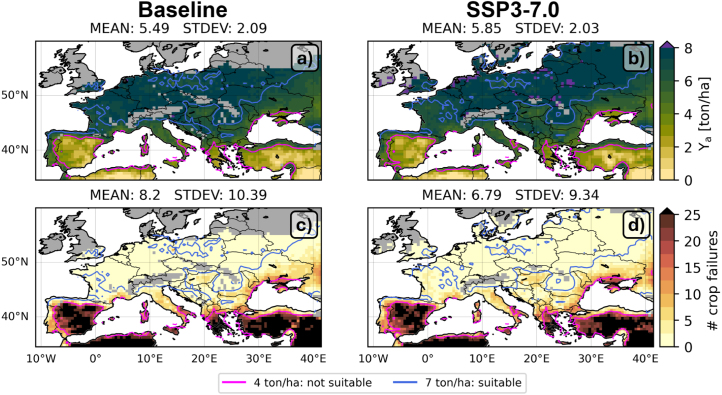
(Top row) 30-year average $Y_{a}$ for the (a) baseline and (b) SSP3-7.0 scenario, with an indication of area that is unsuitable for rainfed maize production (<4 ton/ha), and the profitable area for production (>7 ton/ha). (Bottom row) number of crop failures (<4 ton/ha) during the 30-year period for the (c) baseline and (d) SSP3-7.0 scenario.

The above results focus on relative changes of SSP3-7.0 to baseline simulations over the area where baseline simulations could accumulate enough GDDs. Fig. 7 shows the average baseline and SSP3-7.0 $Y_{a}$ without the spatial crossmasking between the baseline and SSP3-7.0. Unsuitable production areas are marked where the rainfed yield is below 4 ton/ha, and areas of biophysical profitable production are delineated where the yield is larger than 7 ton/ha. The suitable baseline rainfed production area covers most of northern Europe on the mainland and excludes Mediterranean countries (south Spain, south Italy, Greece, North Africa) and most of Turkey, where maize production actually relies on irrigation. In SSP3-7.0, the dominant productive area shifts north with the southernmost boundary in northern France, and new suitable areas in the UK, Ireland and Scandinavian countries where the temperature was a limitation in the baseline. The larger suitable production area for rainfed maize leads to a higher spatial mean $Y_{a}$. However, when crossmasking the data for the baseline and SSP3-7.0 scenario, the SSP3-7.0 $Y_{a}$ remains similar to the baseline (Fig. 1a, Fig. 2).

Evidently, a shift in land use is not trivial. However, a sustainable intensification of agricultural practices (fertility and/or irrigation) on the existing fields within the productive baseline area can counter any loss of the most southern productive agricultural land for rainfed maize in SSP3-7.0, as suggested by the fact that $Y_{w}$ and $Y_{p}$ for SSP3-7.0 are significantly larger than $Y_{a}$ for the baseline (Fig. 4).

In addition to identifying regions that are (un)suitable for rainfed maize production, our results also show when maize grows best in time, unlike most studies that use a preset crop calendar (Jaegermeyr et al., 2021, Franch et al., 2022). For SSP3-7.0, the growing cycle becomes shorter, and the sowing date is earlier, in line with (Supit et al., 2010). The yield is maintained for the shorter growing cycle, because of higher ET${}_{o}$ (Fig. 3), and thus transpiration and biomass production. Parent et al. (2018) argue that exploiting the genetic variability of flowering time for different maize breeds can be used to counteract cycle shortening and maintain or even increase maize production under climate change. Note that the shorter growing cycle in our SSP3-7.0 simulations limits the chances of hitting adverse weather and likely helps to replenish soil moisture after an earlier harvest, allowing more available water at the beginning of the next growing season. Minoli et al. (2022) similarly state that timely adaptation of growing periods could limit or even prevent yield losses. Shorter growing cycles also open up the possibility of growing multiple crops sequentially in the same year, but only if there is enough water and nutrient supply.

### 4.2 Irrigation versus fertility management

Both the elimination of fertility stress through field management, and the elimination of water stress through irrigation, contribute to potentially increased yields in both the baseline and future scenarios. Water-limited areas in the south do not benefit much from the removal of fertility stress, whereas northern areas do. Increasing fertility can largely bridge the $YG_{w}$ in the north. In contrast, water-limited areas evidently benefit from irrigation and northern areas only have a relatively small benefit from eliminating water stress on top of eliminating fertility stress. For northern latitudes, fertility increases the $WP_{ET}$, but irrigation cannot further increase it. In southern countries, $WP_{ET}$ increases with irrigation. Given the available maps of irrigation infrastructure (e.g., Siebert et al., 2005), only a small area in the southern regions is currently able to bridge $YG_{p}$ and reach $WP_{p,ET}$ via irrigation to sustain maize cultivation (Webber et al., 2016). However, this does not take into account water availability, which might be a problem under future scenarios (Haddeland et al., 2014).

**Fig. C.1:**
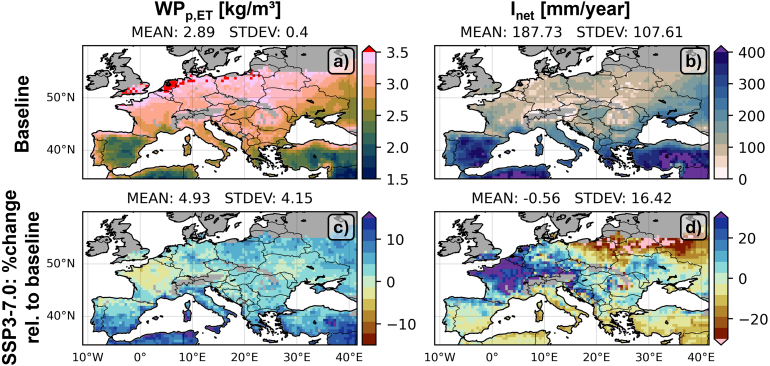
30-year average of (top) baseline $WP_{p,ET}$, and $I_{net}$, (second row) difference between SSP3-7.0 and baseline $WP_{p,ET}$, and $I_{net}$ relative to the respective baseline estimates [relative change, %].

The impact of climate on irrigated maize is not discussed above, but can be inferred from the simulations of $Y_{p}$ (Fig. 4) and $WP_{p,ET}$ for the southern countries in Fig. C.1. Our simulations suggest that $Y_{p}$ will remain stable and $WP_{p,ET}$ will increase for SSP3-7.0. This is in contrast to Yang et al. (2017) who reported decline in $Y_{p}$ and $WP_{p,ET}$ for maize in Portugal for future climate scenarios: additional irrigation decreased their $WP_{p,ET}$. Busschaert et al. (2022) also reported the need for more irrigation under climate change, when assuming a fixed calendar crop. In our case, the earlier and shorter growing season, and the CO${}_{2}$ enriched environment, limit the required net irrigation (Fig. C.1d) and thus increase $WP_{p,ET}$ (Fig. C.1c). Nevertheless, these results should be taken with caution, as the maize crop is only calibrated for rainfed conditions.

### 4.3 Uncertainties

#### 4.3.1 Climate projections

**Fig. 8:**
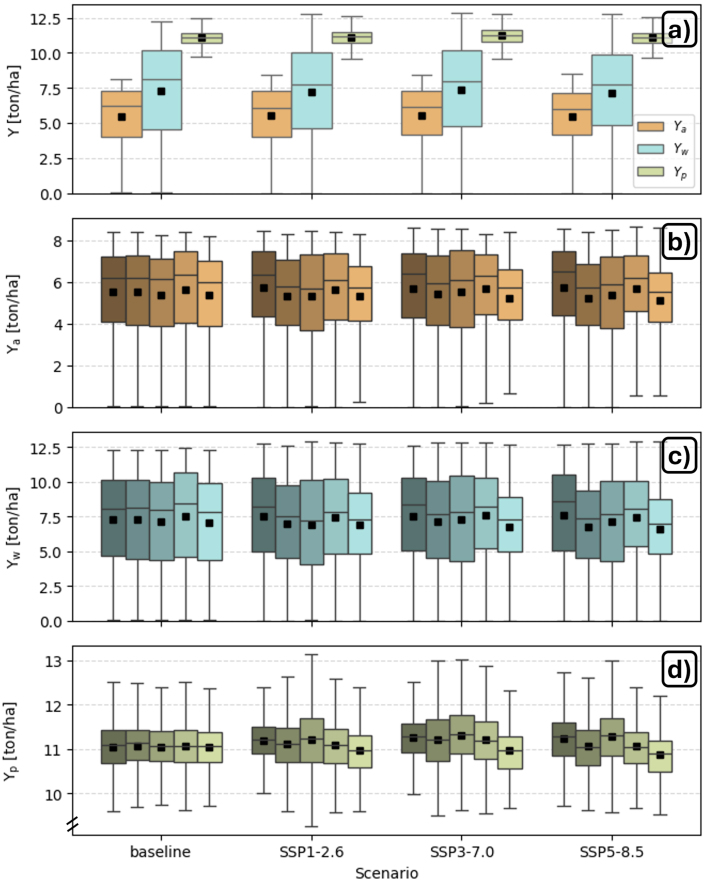
Boxplots showing the spatial distribution of 30-year averaged $Y_{a}$, $Y_{w}$, and $Y_{p}$ for the baseline and 3 climate scenarios. The box edges represent the 25th and 75th percentiles, and the middle line is the median. The squares represent the spatial mean. Outliers are not shown. (a) Boxplots of $Y_{a}$, $Y_{w}$, and $Y_{p}$, where each grid cell contains the median GCM value. Boxplots for (b) $Y_{a}$, (c) $Y_{w}$, and (d) $Y_{p}$ for 5 GCMs, from left to right: GFDL-ESM4, IPSL-CM6A-LR, MPI-ESM1-2-HR, MRI-ESM2-0, and UKESM1-0-LL.

Future scenarios contain uncertainty and sample only a part of possible futures. Fig. 8 shows the variation in GCM-median 30-year averaged $Y_{a}$, $Y_{w}$ and $Y_{p}$ for the baseline and the 3 future climate scenarios, when crossmasked to the same area with enough GDDs to complete the crop cycle. This is essentially the area for the baseline simulation, including unsuitable areas, and not including the expansion of the potential area for biophysical profitable production as discussed above. Fig. 8a summarizes that the various types of yield (and thus the yield gaps) for different climate scenarios remain similar to the baseline over Europe, due to adaptation with temperature-dependent sowing dates and growing season lengths, and adaptive amounts of irrigation water for $Y_{p}$. The impact of the various climate scenarios is much smaller than eliminating fertility or water limitations. The spatial variation in $Y_{a}$ is high because areas unsuitable for rainfed maize production are included. There is an upper limit to $Y_{a}$ due to climatic, crop cultivar and management constraints. The spatial variation systematically increases for $Y_{w}$ because avoiding fertility stress helps most for the higher latitudes, where enough water is available, but only marginally helps in the dry southern regions. The spatial variation in $Y_{p}$ is minimal by design, because the spatial variation in fertility and water limitations is removed.

Fig. 8b and c show $Y_{a}$ and $Y_{w}$ for each of the 5 GCMs individually. For both, the relative variation across the GCMs and climate scenarios is similar ($Y_{a}$ and $Y_{w}$ are mostly driven by the GCM climate). Only for the most severe climate scenario SSP5-8.5, $Y_{a}$ sightly decreases for most GCMs, except for GFDL-ESM4 with wetter conditions. By contrast, $Y_{p}$ (Fig. 8d) remains fairly stable, except for UKESM1-0-LL, which has a lower $Y_{p}$. This suggests that there is a slight increase in $YG_{p}$ for the most severe scenario for some GCMs.

As in Busschaert et al. (2022), the differences between the GCMs are as large as the differences between climate scenarios, highlighting the importance of using several GCMs when assessing future climate impacts (O’Loughlin, 2021). The various GCMs might have different timings for heat waves and droughts, which may have a great impact on the yield, especially if they occur during the flowering stage. In addition to climate boundary conditions, the groundwater table is an important lower boundary condition for maize simulation (Zhao et al., 2020, Pinke et al., 2020), which is not included in this study, but could be recommended for future research. Finally, it is expected that the effects of climate change will be larger (and more uncertain) in the far-future (e.g., the end of the century) compared to the near-future period selected in this study (2030–2059), as demonstrated by Busschaert et al. (2022) for SSP3-7.0 and SSP5-8.5.

#### 4.3.2 Crop model

The structure of the crop model (Kimball et al., 2023, Ran et al., 2020) and the parameterization of the soil fertility, and crop (Vanuytrecht et al., 2014, Sandhu and Irmak, 2019, Lu et al., 2021) also introduce limitations and uncertainties. Numerous model developments are still expected in their ability to simulate the phenology of the crop (e.g. improved GDD calculation; Zhou and Wang, 2018) and the soil water balance (e.g. use of the Richards equation). Müller et al. (2024) highlights how crop models differ in their sensitivity to the drivers of crop yield, i.e., carbon dioxide concentrations, temperature, water, and nitrogen. This model sensitivity may deviate from the real plant sensitivities, which could be determined in controlled climate chambers (Boote et al., 2018, Rineau et al., 2019).

Although our AquaCrop simulations fall within the range of GYGA data and also agree with independent model simulations in the literature (Schils et al., 2018, Mialyk et al., 2022), various assumptions remain uncertain. Our assumption of one generic maize cultivar across the domain has limitations because, in reality, hundreds of cultivars are used in Europe (Parent et al., 2018). They vary greatly in the length of growing stages, time of flowering, and tolerance to drought stress, which are all determinant factors of yield production. By using region specific variants, the yield estimates are expected to be higher because variants are locally selected to maximize the production. But given the resolution of this study and the uncertainty in future cultivar usage and development, a fixed crop variant constitutes a reasonable assumption.

Similarly, a uniform soil fertility stress over the domain could be improved in future studies. Further work is needed on soil fertility stress maps before including those in this type of study. Also note that the GYGA $Y_{a}$ records do not necessarily agree with other literature values, because field-based dry yield data may pertain to a specific year or location within a country, or may not be expressed for the same level of moisture. For example, Stricevic et al. (2011) report 8 to 14 ton/ha in Serbia (east Europe), whereas GYGA reports 6 ton/ha.

Although the adaptive sowing date is a novelty in large-scale climate impact assessments, basing it solely on a temperature criterion might be a limitation. This can be enhanced in future research by e.g. including a moisture-based criteria, because sowing maize in dry conditions is commonly avoided (Fang et al., 2021).

#### 4.3.3 Interannual variability

This paper focuses on multiyear averaged yields and yield gaps, but in reality yield has a multitemporal variability: this can be caused by long-term persistent factors across the years and annually varying non-persistent limiting factors (Hoffmann et al., 2018). Literature suggests that with climate change, more extreme events are expected to lead to more crop yield failures (de S. Nóia-Júnior et al., 2025), and more years with extremes in $YG_{w}$ (Lopes, 2022), important for rainfed maize. By contrast, $YG_{p}$ should remain more stable over the years (Farmaha et al., 2016), and this is relevant for irrigated maize. Fig. B.1 indeed confirms an increased temporal variation in yield for SSP3-7.0. However, if a crop failure is defined as a yield of less than 4 ton/ha, then Fig. 7 shows no substantial increase in crop failures and a shift of the production area to the north could even decrease the chance of a crop failure.

#### 4.3.4 Unconsidered factors

The yield losses in AquaCrop are primarily driven by water shortages, but in reality other factors should be considered, such as biotic stresses (pests, diseases) and extreme events (floods, wind throw, heat waves). These factors will reduce the yields and introduce a larger interannual variability (Section 4.3.3). Although pests and diseases still claim 10%–16% of the global harvest, their evolution with climate change is uncertain and highly dependent on crop types (Chakraborty and Newton, 2011). In addition, the effect of extreme climate events is difficult to quantify, because (i) GCMs are not designed to accurately represent extreme events like heavy rains and winds because these events are highly localized and punctual (Masud et al., 2021), and (ii) the crop models, used to make these yield projections, only have a poor representation of the negative effects in wet conditions, compared to their ability to simulate drought stress (van der Velde et al., 2012). These biotic stresses and extreme events, were shown to have a milder (more localized) effect (Kim et al., 2023, Lesk et al., 2016).

Another level of uncertainty is the future socioeconomic evolution (national wealth, farm size, policy support) that can have a significant contribution to the closure of the yield gap (Schils et al., 2018). Future changes in crop parameters for new cultivars and biotechnological developments (Parent et al., 2018, Chen et al., 2012) and adaptive farmer practices (Baum et al., 2019) can have the potential to counteract negative climate impacts.

## 5 Conclusions

Maize crop production is controlled by climate conditions and farmers’ practices, such as fertility management and irrigation. To assess whether and how Europe, as an important grain maize producer, can maintain or increase its grain maize production in the future, we simulate yields and yield gaps for the current and future climate, with and without fertility and water limitations. The water-limited yield gap is defined as the relative difference between actual farmers’ yield ($Y_{a}$) and water-limited yield without any fertility stress ($Y_{w}$). The potential yield gap refers to the relative difference between $Y_{a}$ and potential yield $Y_{p}$ without any water or fertility stress, i.e. by including irrigation. The crop simulations are performed spatially at a 0.5°resolution, using AquaCrop v7.2 forced with climate simulations from ISIMIP3 for a baseline (1985–2014) and future period (2030–2059), and using 5 different global climate models (GCMs). The generic grain maize crop is defined with a temperature-dependent sowing date and temperature-dependent growing stages, which allows for climate adaptation of the growing cycle, in contrast to some earlier climate impact assessments.

The results show that a warmer climate generally leads to earlier sowing dates and shorter growing seasons. On average over Europe, climate change has only a small impact on $Y_{a}$, $Y_{w}$ and $Y_{p}$, leading to small changes in yield gaps, due to adaptation of the growing season and some CO${}_{2}$ fertilization. For northern latitudes, both the yield and water productivity increase for rainfed maize, whereas the central latitudes lose some rainfed yield. Furthermore, warmer climate conditions allow to expand and move the area of profitable crop production north for rainfed maize. The future $Y_{p}$ projections are also similar to the baseline, but they show an increased water productivity, again due to an earlier and shorter growing season.

The gain in yield through the elimination of fertility or water limitations is larger than the impact of climate change. When excluding the most southern region (which is not suitable for the production of rainfed maize), the average yield increases by 1.5 ton/ha by removing fertility stress and it further increases by 2 ton/ha through additional irrigation. However, optimizing fertility alone is only helpful for northern latitudes without water limitations. In contrast, optimal water supply via irrigation cannot add much value in the north and mostly benefits southern latitudes, where water supply is uncertain. It is important to note that the yield simulations mainly account for drought stress and do not consider other climatic losses, such as flooding and wind throw.

To convert these findings into practical socio-economic applications, the yield per unit area needs to be weighted by the area dedicated to agriculture to produce total amounts of food production or economic return. The area for maize production is determined not only by soil texture and other landscape factors but will also vary with climate and policies, highlighting the need for land-use strategies that prioritize sustainable expansion and efficient allocation of cropland. Furthermore, future crop cultivars are likely to also adapt to the climate, underscoring opportunities for targeted investment in irrigation infrastructure and breeding programs. These factors should be addressed in future interdisciplinary climate assessment studies to better inform agricultural policy and long-term planning.

## CRediT authorship contribution statement

**Louise Busschaert:** Writing – review & editing, Visualization, Software, Methodology, Formal analysis. **Vincent Deketelaere:** Writing – review & editing, Software, Methodology, Formal analysis, Conceptualization. **Wim Thiery:** Writing – review & editing. **Dirk Raes:** Writing – review & editing, Software, Methodology, Conceptualization. **Gabriëlle J.M. De Lannoy:** Writing – original draft, Supervision, Resources, Methodology, Funding acquisition, Conceptualization.

## Funding

The computer resources and services were provided by the High Performance Computing system of the Vlaams Supercomputer Center, funded by the 10.13039/501100003130Research Foundation Flanders FWO, Belgium and the Flemish Government (incl. Storage4Climate collaborative grant). This work received support from project C14/21/057 ofKU Leuven, and CROPWAVES of the Belgian Science Policy (Belspo). Louise Busschaert is funded by 10.13039/501100003130FWO, Belgium grant 1158423N. Wim Thiery acknowledges funding from the 10.13039/501100000781European Research Council (ERC) under the European Union’s Horizon Framework research and innovation programme (grant agreement No 101124572; ERC Consolidator Grant ‘LACRIMA’).

## Declaration of competing interest

The authors declare that they have no known competing financial interests or personal relationships that could have appeared to influence the work reported in this paper.

## Figures and Tables

**Table A.1:** AquaCrop crop parameters of generic rainfed maize for Europe.

Crop Parameter	Value	Unit/Description
AquaCrop Version	7.2	August 2024
Crop type	2	Fruit/grain producing crop
Sowing	1	Crop is sown
Cycle determination	0	By growing degree-days
ET${}_{o}$ adjustment	1	Adjusted soil water depletion factors
Base temperature	8.0	°C (Development does not progress below this)
Upper temperature	30.0	°C (No increase above this)
Crop cycle length	1122	Growing degree-days
Canopy expansion (p-exp)	0.14/0.72	Upper/Lower threshold for soil water depletion factor
Water stress shape factor (canopy expansion)	2.9	0.0 = Straight line
Stomatal control (p-sto)	0.69	Upper threshold for depletion fraction
Water stress shape factor (stomatal control)	6.0	0.0 = Straight line
Canopy senescence (p-sen)	0.69	Upper threshold for soil water depletion
Water stress shape factor (senescence)	2.7	0.0 = Straight line
Dormant period ET${}_{o}$ sum	50	Permanent wilting threshold
Pollination (p-pol)	0.80	Upper threshold for soil water depletion
Anaerobiotic point	5	Vol% at deficient aeration
Soil fertility stress calibration	30	Considered for calibration (%)
Shape factor (canopy expansion)	2.55	Response to soil fertility stress
Shape factor (maximum canopy)	1.38	Response to soil fertility stress
Shape factor (water productivity)	−1.18	Response to soil fertility stress
Shape factor (canopy decline)	4.7	Response to soil fertility stress
Minimum pollination temperature	10	°C (Cold stress threshold)
Maximum pollination temperature	40	°C (Heat stress threshold)
Full transpiration requirement	12.0	Growing degree days (°C-day)
Soil salinity effect start	2	dS/m (EC of soil saturation)
Soil salinity growth end	10	dS/m (EC of soil saturation)
Salinity stress distortion (CC)	25	Distortion due to salinity (%)
Stomata stress response	100	Response to ECsw (%)
Crop coefficient (KcTr,x)	1.15	Before senescence
Decline in crop coefficient	0.300	%/day
Min rooting depth	0.30	m
Max rooting depth	1.00	m
Root zone expansion shape factor	13	
Max root extraction (top)	0.048	m${}^{3}$/m${}^{3}$ soil/day (top quarter)
Max root extraction (bottom)	0.012	m${}^{3}$/m${}^{3}$ soil/day (bottom quarter)
Late season evaporation reduction	50	Effect of canopy cover (%)
Seedling surface cover	6.50	cm${}^{2}$ at 90% emergence
Plant canopy size (day 1)	6.50	cm${}^{2}$ (Re-growth)
Plants per hectare	75 000	Number/ha
Canopy growth coefficient (CGC)	0.12201	Increase in fraction soil cover/day
Self-thinning (CCx decline)	−9	Not applicable
Max canopy cover (CCx)	0.96	Fraction soil cover
Canopy decline coefficient (CDC)	0.09004	Fraction per day
Days to emergence	6	Calendar days (not used)
Days to max rooting depth	82	Calendar days (not used)
Days to start senescence	120	Calendar days (not used)
Days to maturity	152	Calendar days (not used)
Days to flowering	66	Calendar days (not used)
Length of flowering stage	13	Days (not used)
Determinacy	1	Linked with flowering
Potential fruits excess	50	%
Harvest Index start (flowering)	81	Days
Water productivity (WP*)	33.7	g/m${}^{2}$, normalized for ET${}_{o}$ and CO${}_{2}$
Yield formation productivity	100	% of WP*
CO${}_{2}$ response strength	50	%
Reference Harvest Index (HIo)	48	%
Pre-flowering HI increase	0	% due to water stress
Positive HI impact (restricted growth)	7.0	
Negative HI impact (stomatal closure)	3.0	During yield formation
Max HI increase	15	%
GDD to emergence	20	°C
GDD to max rooting depth	396	°C
GDD to senescence start	788	°C
GDD to maturity	1122	°C
GDD to flowering	276	°C
Flowering stage length	98	°C
CGC for GDD	0.026470	Increase in fraction soil cover per GDD
CDC for GDD	0.008601	Decline in fraction per GDD
GDD for Harvest Index	783	Building-up during yield formation
Dry matter (fresh yield)	85	%
Rooting depth (year 1)	0.00	m (For regrowth)
Sowing year	1	1st year, for regrowth
Assimilate transfer	0	Not considered
Season-end assimilate storage	0	Days
Transfer to roots (end of season)	0	%
Stored assimilates transfer	0	% (Next season)

## Data Availability

AquaCrop v7.2 is open source available at https://github.com/KUL-RSDA/AquaCrop, and the Python wrapper for spatial simulations can be found at https://github.com/KUL-RSDA/RegionalAC_Py. The simulation input (climate and soil files) are identical to the setup of (Busschaert et al., 2022) and can be retrieved from https://zenodo.org/records/6760977. The GYGA yield data can be retrieved from https://www.yieldgap.org/. The yield output is available on Zenodo (Busschaert et al., 2025).

## Appendix A Crop file for rainfed grain maize

The maize crop is parameterized using (i) default conservative parameters (i.e., independent of time, location or field management practices) taken from the default maize crop of AquaCrop (Hsiao et al., 2009), resulting from intensive research on maize in California (US), and (ii) lengths of development stages in calendar days that are roughly in line with European maize cultivars. Specifically, 31, 39, 50 and 32 days are chosen for the initial, development, mid-season and late season phases, respectively, based on literature for Europe, and Allen et al. (1998) (their Table 11). The flowering is set at 66 days after sowing, and takes 13 days, following (Doorenbos et al., 1979). Note that maize is a determinant crop that stops growing when it reaches the flowering stage. The crop stages are thus initially estimated in calendar days. The file mode is then switched from calendar days to growing-degree-days (GDD), using representative meteorological input for Belgium (2016), taken from ISIMIP3a. The maximum effective rooting depth is set to 1 m and the crop coefficient for maximum crop transpiration (when the canopy is complete, Kc${}_{Tr,x}$) to 1.15 [-], in line with Allen et al. (1998) (their Table 17).

(See Table A.1.)

## Appendix B Variation in maize yield

See Fig. B.1.

## Appendix C Climate impact on $WP_{p,ET}$ and irrigation requirements

See Fig. C.1.
